# Association Between Anticoagulation Outcomes and Venous Thromboembolism History in Chronic Thromboembolic Pulmonary Hypertension

**DOI:** 10.3389/fcvm.2021.628284

**Published:** 2021-05-21

**Authors:** Yong-Jian Zhu, Yu-Ping Zhou, Yun-Peng Wei, Xi-Qi Xu, Xin-Xin Yan, Chao Liu, Xi-Jie Zhu, Zi-Yi Liu, Kai Sun, Lu Hua, Xin Jiang, Zhi-Cheng Jing

**Affiliations:** ^1^Department of Cardiology, Peking Union Medical College Hospital, Chinese Academy of Medical Sciences and Peking Union Medical College, Beijing, China; ^2^Department of Pulmonary Vascular Disease and Thrombosis Medicine, State Key Laboratory of Cardiovascular Disease, National Center for Cardiovascular Diseases, FuWai Hospital, Chinese Academy Medical Sciences and Peking Union Medical College, Beijing, China

**Keywords:** anticoagulation, chronic thromboembolic pulmonary hypertension, venous thromboembolism, bleeding, recurrence

## Abstract

**Background:** The association between anticoagulation outcomes and prior history of venous thromboembolism (VTE) in chronic thromboembolic pulmonary hypertension (CTEPH) has not been established. This study aimed to compare the efficacy and safety of anticoagulation treatment in CTEPH patients with and without prior history of VTE.

**Methods:** A total of 333 CTEPH patients prescribed anticoagulants were retrospectively included from May 2013 to April 2019. The clinical characteristics were collected at their first admission. Incidental recurrent VTE and clinically relevant bleeding were recorded during follow-up. The Cox proportional regression models were used to identify potential factors associated with recurrent VTE and clinically relevant bleeding.

**Results:** Seventy patients (21%) without a prior history of VTE did not experience recurrent VTE during anticoagulation. Compared to CTEPH patients without a prior history of VTE, those with a prior history of VTE had an increased risk of recurrent VTE [2.27/100 person-year vs. 0/100 person-year; hazard ratio (HR), 8.92; 95% confidence interval (CI), 1.18–1142.00; *P* = 0.029] but a similar risk of clinically relevant bleeding (3.90/100 person-year vs. 4.59/100 person-year; HR, 0.83; 95% CI, 0.38–1.78; *P* = 0.623). Multivariate Cox analyses suggested that a prior history of VTE and interruption of anticoagulation treatments were significantly associated with an increased risk of recurrent VTE, while anemia and glucocorticoid use were significantly associated with a higher risk of clinically relevant bleeding.

**Conclusions:** This study is the first to reveal that a prior history of VTE significantly increases the risk of recurrent VTE in CTEPH patients during anticoagulation treatment. This finding should be further evaluated in prospective studies.

## Introduction

Chronic thromboembolic pulmonary hypertension (CTEPH), classified within group 4 pulmonary hypertension (PH), is a rare and life-threatening disease if left untreated (1, 2). It has been well-established that CTEPH could be a late complication of acute pulmonary embolism (PE). However, the prevalence of a previous PE history among CTEPH patients varied from only 15.3% in a Japanese cohort to 74.8% in a western registry (1, 3). The absence of previous venous thromboembolism (VTE) suggests some undiscovered causes, possibly contributing to the pathogenesis or progression of CTEPH.

Considering the close relationship between thromboembolic disorders and the development of CTEPH, anticoagulation is always regarded as the cornerstone of the current treatment algorithm of CTEPH. Life-long anticoagulation treatment is recommended by current guidelines for CTEPH patients in the absence of contraindications (2, 4). Although warfarin was recommended as the treatment of choice, direct oral anticoagulants (DOACs) have been alternatively used considering their proven therapeutic effects in VTE and administrative convenience over warfarin. Recently, several studies have reported the efficacy and safety of anticoagulants including warfarin and rivaroxaban in CTEPH patients (5–9). However, these studies failed to identify the differences in anticoagulation outcomes between CTEPH patients with and without prior VTE history. Evidence about the benefit-to-risk balance for anticoagulation treatments in CTEPH patients without prior VTE history is still limited. Moreover, few studies have investigated the risk factors of recurrent VTE and bleeding events over long-term anticoagulation treatment in CTEPH patients.

Therefore, this study aimed to compare the efficacy and safety of anticoagulation treatment between CTEPH patients with and without prior VTE history and to investigate the risk factors of recurrent VTE and clinically relevant bleeding in CTEPH patients.

## Methods

### Participants and Data Collection

All consecutive patients diagnosed with CTEPH in the Department of Pulmonary Vascular Disease and Thrombosis Medicine, FuWai Hospital from May 2013 to April 2019 were recruited retrospectively. The PH was confirmed by right heart catheterization. The diagnosis of CTEPH in this study was based on the recommendations of international guidelines (2, 4) and confirmed by a multidisciplinary team specialized in pulmonary hypertension, including senior cardiologists, radiologists, and surgeons. The details were described in our previous study (10). Patients refusing participation or with contraindications for anticoagulation treatment, such as frequent hemoptysis, uncontrolled active bleeding, and other conditions with remarkably high risk of bleeding, were excluded from this study.

Patients' demographic information, prior VTE history, risk factors of CTEPH development, hemodynamic parameters, comorbidities, and treatments were collected on their first admission. All patients were routinely assessed for potential risk factors of CTEPH including recurrent VTE, antiphospholipid syndrome (APS), splenectomy, pacemaker implantation, chronic inflammatory disease, known hypothyroidism, and ventriculo-atrial shunt. Prior VTE history before the diagnosis of CTEPH was considered as previously diagnosed PE/deep vein thrombosis (DVT) confirmed by objective imaging modalities (compression ultrasound, computed tomography pulmonary angiography, lung ventilation-perfusion scan, or pulmonary angiography). The protocol was approved by the Institutional Ethics Review Board of FuWai Hospital, and all patients gave written informed consent.

### Anticoagulation Regimens

The types of anticoagulants prescribed on admission were at the physicians' discretion and of the patients' choice. Patients administrated with warfarin routinely received an initial dose of 3 mg once daily, following appropriate adjustment of warfarin dose to maintain the international normalized ratio to the targeted range (2.0–3.0). Three DOACs available in China are rivaroxaban, dabigatran, and apixaban. DOACs were prescribed at a fixed dose (20 mg once daily for rivaroxaban, 110 mg twice daily for dabigatran, and 5 mg twice daily for apixaban). For patients with a potential risk of bleeding, the dose of DOACs was decreased at the discretion of the physicians. During anticoagulation treatment, when bleeding events occurred, the decision about whether and when to discontinue and resume the anticoagulant regimen and dosage was made by physicians.

### Follow-Up and Outcome Assessment

All patients were followed up *via* clinic visits or by phone or internet interviews, and the observation period was from the initial diagnosis of CTEPH to November 2019 or the latest visit. The effectiveness outcome was the time from diagnosis of CTEPH to the occurrence of the first recurrent VTE, which was defined as a symptomatically new onset of PE or DVT confirmed by objective imaging modalities from medical records. The imaging data were reassessed by a senior cardiologist and radiologist collectively, and the serial imaging of patients was comparatively analyzed as appropriate. The safety outcome was the time from diagnosis of CTEPH to the occurrence of clinically relevant bleeding, which was defined as a composite outcome of major bleeding and non-major clinically relevant bleeding. The major bleeding should be overt and classified in line with the International Society on Thrombosis and Haemostasis (ISTH) criteria (11). Briefly, major bleeding should meet one of the following conditions: (i) bleeding in a critical site, (ii) bleeding causing a reduction of 2 g/dL or more in hemoglobin levels or leading to a transfusion of 2 or more units of packed red blood cells or whole blood, and (iii) contributing to death. Non-major clinically relevant bleeding was defined as non-major bleeding associated with anticoagulant interruption of more than 7 days or emergency hospital admission.

### Statistical Analysis

Descriptive data were presented as numbers (percentage) for categorical variables, mean (standard deviation) for continuous variables that were normally distributed, and median [interquartile range (IQR), 25–75th percentiles] for continuous variables, which were non-normally distributed. Person-year for each participant was calculated from the date of diagnosis until the date of last visit or end of the study.

The chi-square test, independent *t*-test, and Mann–Whitney *U*-test were used as appropriate. The incidence rate was calculated as the number of events divided by person-year. The log-rank test was adopted to compare the cumulative incidence of the first occurrence of recurrent VTE and clinically relevant bleeding. The Cox proportional regression models were used to identify the factors associated with recurrent VTE and clinically relevant bleeding. The variables in the final multivariable model included those which showed an association with the outcome at a level of 0.05 in univariate analysis. Additionally, age, sex, and anticoagulation treatments were also included based on clinical concerns. If a model did not converge due to a low number of events, the model was fitted using Firth's penalized likelihood approach (12).

All statistical analyses were performed using R version 3.6.0 (R Core Team, Vienna, Austria) and IBM SPSS Statistics for Windows, Version 22.0 (Armonk, NY: IBM Corp). A two-sided *P* < 0.05 was considered statistically significant.

## Results

### Baseline Characteristics

In total, 333 patients with CTEPH were included in baseline characteristics analysis (Table 1). The mean age of these patients was 53.5 ± 14.6 years, and there was an approximately equal gender distribution (female, 45.9%). The majority of patients (79.0%) had a confirmed history of VTE (PE and/or DVT). DOACs were prescribed as first-line anticoagulants in most of the patients (74.2%), and all other patients were treated with warfarin. Rivaroxaban was predominantly used in 95.5% of patients in the DOACs group; dabigatran and apixaban were prescribed in 3.6% and 0.8% of patients, respectively (Supplementary Table 1). Both the two patients who received apixaban were treated with a half-dose (2.5 mg twice daily) instead of the recommended dosage because of prior bleeding. Compared to the patients with prior VTE, patients without prior VTE were more likely to be female and presented with more compromised pulmonary hemodynamics. Moreover, they had less APS and more warfarin treatment.

**Table 1:** Demographic and clinical characteristics of included CTEPH patients.

	**Total (*n* = 333)**	**Prior VTE (*n* = 263)**	**No prior VTE (*n* = 70)**	***P*-value**
**Demographic variables**				
Age, years, mean (SD)	53.5 ± 14.6	53.1 ± 13.1	55.1 ± 12.5	0.259
Female sex, *n* (%)	153 (45.9)	109 (41.4)	44 (62.9)	0.001
Blood group non-O, *n* (%)	271 (81.4)	214 (81.4)	57 (81.4)	0.991
**Prior VTE history**				
PE, *n* (%)	232 (69.7)	232 (88.2)	0 (0)	<0.001
DVT, *n* (%)	159 (47.7)	159 (60.5)	0 (0)	<0.001
**Comorbid conditions**				
APS, *n* (%)	24 (7.2)	24 (9.1)	0 (0)	0.009
Pacemaker, *n* (%)	0 (0)	0 (0)	0 (0)	–
Splenectomy, *n* (%)	1 (0.3)	1 (0.4)	0 (0)	1.000
Active cancer, *n* (%)	3 (0.9)	2 (0.8)	1 (1.4)	0.509
Renal insufficient, *n* (%)	9 (2.7)	8 (3.0)	1 (1.4)	0.745
Coronary artery disease, *n* (%)	24 (7.2)	15 (5.7)	5 (7.1)	0.867
Ischemic stroke, *n* (%)	26 (7.8)	21 (8.0)	5 (7.1)	0.816
Hypertension, *n* (%)	87 (26.1)	68 (25.9)	19 (27.1)	0.828
Diabetes mellitus, *n* (%)	12 (3.6)	7 (2.7)	5 (7.1)	0.154
Anemia, *n* (%)	28 (8.4)	22 (8.4)	6 (8.6)	0.956
**Clinical variables**				
WHO function class (III-IV), n (%)	210 (63.1)	165 (62.7)	45 (63.4)	0.811
NT-proBNP, pg/mL, median (IQR)	1,208 (396–2,863)	1,309 (359–3,122)	1,015 (383–2,256)	0.320
D-dimer, ng/mL, median (IQR)	403 (170–870)	365 (170–870)	450 (150–865)	0.772
**Hemodynamics, median (IQR)**				
RAP, mm Hg, median (IQR)	7 (4–10)	7 (1, 4–10)	8 (5–10)	0.114
mPAP, mm Hg, median (IQR)	50 (40–59)	48 (39–59)	52 (46–58)	0.021
PAWP, mm Hg, median (IQR)	10 (7–11)	10 (7–11)	11 (8–12)	0.025
CI, L min^−1^ m^−2^, median (IQR)	2.5 (2.1–2.9)	2.5 (2.1–2.9)	2.5 (2.0–2.8)	0.530
PVR, Wood units, median (IQR)	8.8 (6.0–12.2)	8.6 (5.9–11.7)	9.9 (6.9–13.1)	0.005
**Concomitant treatments**				
DOACs, *n* (%)	247 (74.2)	202 (76.9)	45 (64.3)	0.033
PH-targeted drugs, *n* (%)	312 (92.7)	249 (94.7)	63 (90.0)	0.249
Glucocorticoids, *n* (%)	15 (4.5)	12 (4.6)	3 (4.3)	1.000
PEA, *n* (%)	67 (20.1)	59 (22.4)	8 (11.4)	0.041
BPA, *n* (%)	115 (34.5)	87 (35.7)	28 (42.4)	0.313
IVC filter, *n* (%)	48 (14.4)	47 (17.9)	1 (1.4)	0.001

*CTEPH, chronic thromboembolic pulmonary hypertension; VTE, venous thromboembolism; PE, pulmonary embolism; DVT, deep venous thrombosis; APS, antiphospholipid syndrome; NT-proBNP, N-terminal pro-B-type natriuretic peptide; RAP, right atrial pressure; mPAP, mean pulmonary artery pressure; PAWP, pulmonary artery wedge pressure; CI, cardiac index; PVR, pulmonary vascular resistance; DOACs, direct oral anticoagulants; PH, pulmonary hypertension; PEA, pulmonary endarterectomy; BPA, balloon pulmonary angioplasty; IVC, inferior vena cava*.

### The Incidences of Outcome Events

Of all included patients, seven were lost to follow-up during a median period of 25.0 [interquartile range (IQR), 11.9–46.1] months (Supplementary Figure 1). We identified a total of 14 episodes (13 patients) of recurrent VTE, including 6 episodes of PE and 8 episodes of DVT, with an incidence of 1.69/100 person-year for recurrent VTE in CTEPH patients. Thirteen first onset events were recorded for time-to-event analysis, and the median duration of first recurrent VTE since diagnosis was 19.3 (10.8–32.9) months.

Forty-seven clinically relevant bleeding events (1 episode in 23 patients, 2 episodes in 7 patients, 3 episodes in 2 patients, and 4 episodes in 1 patient) were observed during follow-up, yielding an incidence of clinically relevant bleeding and major bleeding of 5.69/100 person-year and 1.69/100 person-year, respectively. Among them, 33 first onset events were recorded for time-to-event analysis. The proportion of detailed hemorrhagic sites are shown in Supplementary Figure 2, and the most common clinically relevant bleeding events were hemoptysis (34%) and gastrointestinal bleeding (29.8%). The types of 15 major bleeding events were six gastrointestinal bleeding, four menorrhagias, three hemoptyses, one intracranial bleeding, and one intramuscular bleeding. Of them, one patient died of huge hemoptysis, and three patients received blood transfusions.

### Prior VTE History and Anticoagulation Outcome

**Figure 1:**
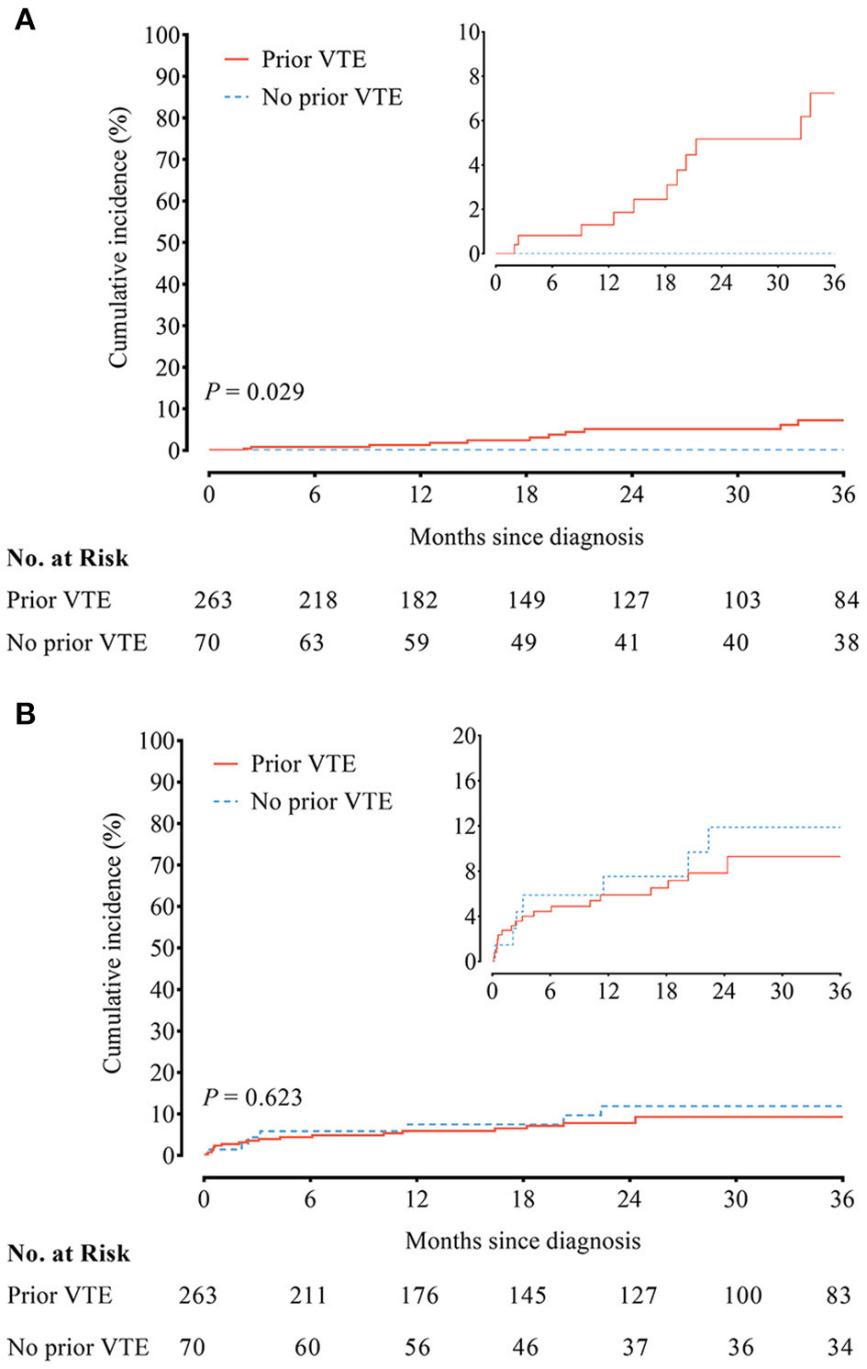
**(A)** Recurrent venous thromboembolism (VTE) and **(B)** clinically relevant bleeding between chronic thromboembolic pulmonary hypertension (CTEPH) patients with and without a prior history of VTE.

No recurrent VTE was observed in 70 CTEPH patients without prior VTE history. In contrast, the incidence of recurrent VTE in CTEPH patients with prior VTE history was 2.27/100 person-year. Compared to CTEPH patients without prior VTE history, those with prior VTE history had an increased risk of recurrent VTE (HR, 8.92; 95% CI, 1.18–1142.00; *P* = 0.029) (Figure 1A and Supplementary Table 2). Twelve episodes of clinically relevant bleeding events were recorded in CTEPH patients with prior VTE history vs. 35 in CTEPH patients without prior VTE history. There were similar incidences of clinically relevant bleeding between CTEPH patients with and without prior VTE history (3.90/100 person-year vs. 4.59/100 person-year), with the HR of 0.83 (95% CI, 0.38–1.78; *P* = 0.623) (Figure 1B and Supplementary Table 3).

### DOACs vs. Warfarin

**Figure 2:**
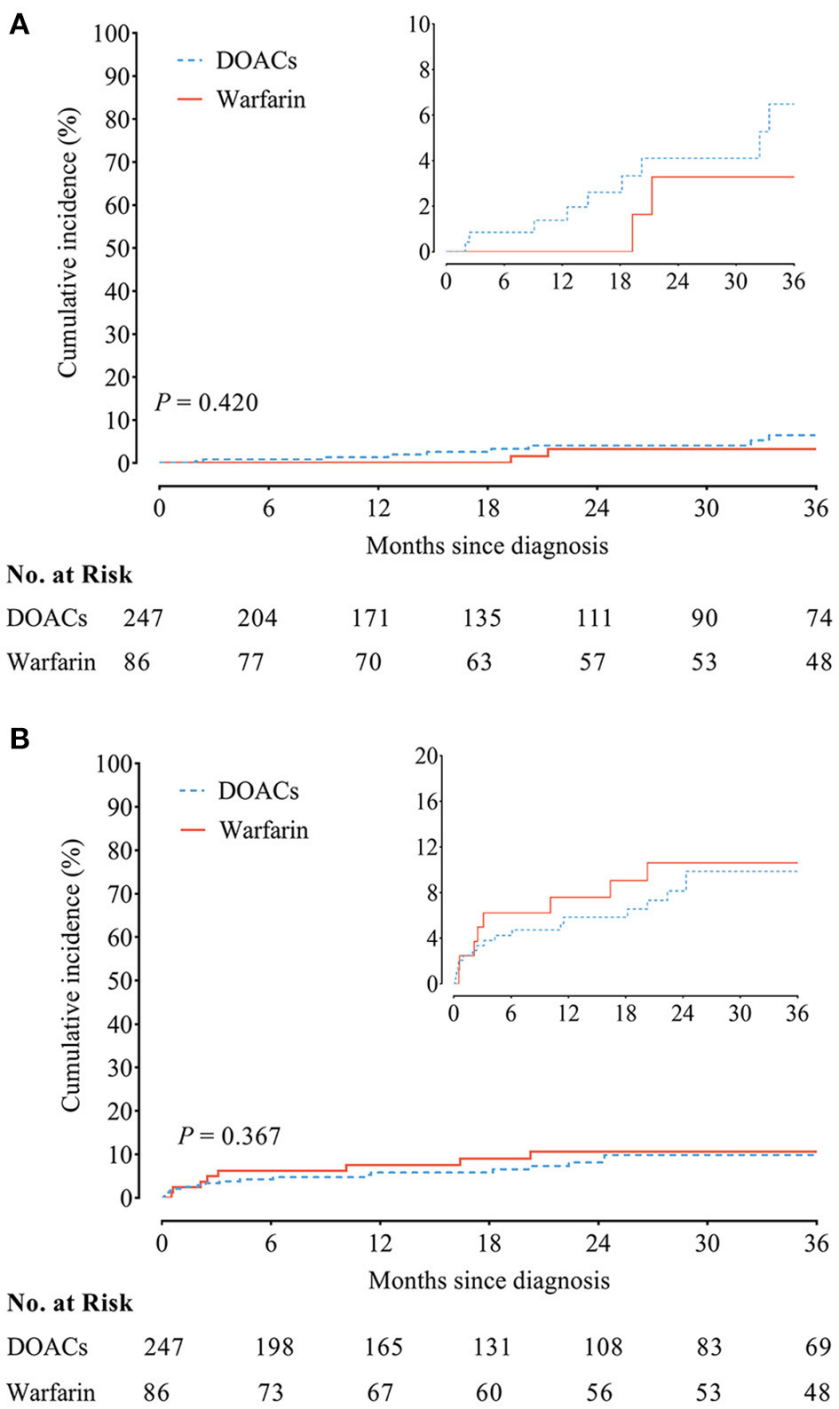
**(A)** Recurrent venous thromboembolism (VTE) and **(B)** clinically relevant bleeding between chronic thromboembolic pulmonary hypertension (CTEPH) patients treated with direct oral anticoagulants (DOACs) and warfarin.

The incidence of recurrent VTE was 1.84/100 person-year in patients receiving DOACs vs. 1.05/100 person-year in patients receiving warfarin, accompanied with similar cumulative incidence curves of recurrent VTE (HR, 1.71; 95% CI, 0.47–6.29; *P* = 0.420) (Figure 2A). In addition, the incidence of clinically relevant bleeding was 2.83/100 person-year as compared with 1.12/100 person-year in patients receiving DOACs and warfarin, yielding similar cumulative incidence curves (HR, 0.72; 95% CI, 0.35–1.47; *P* = 0.367) (Figure 2B).

### Risk Factors Associated With Outcome Events

**Figure 3:**
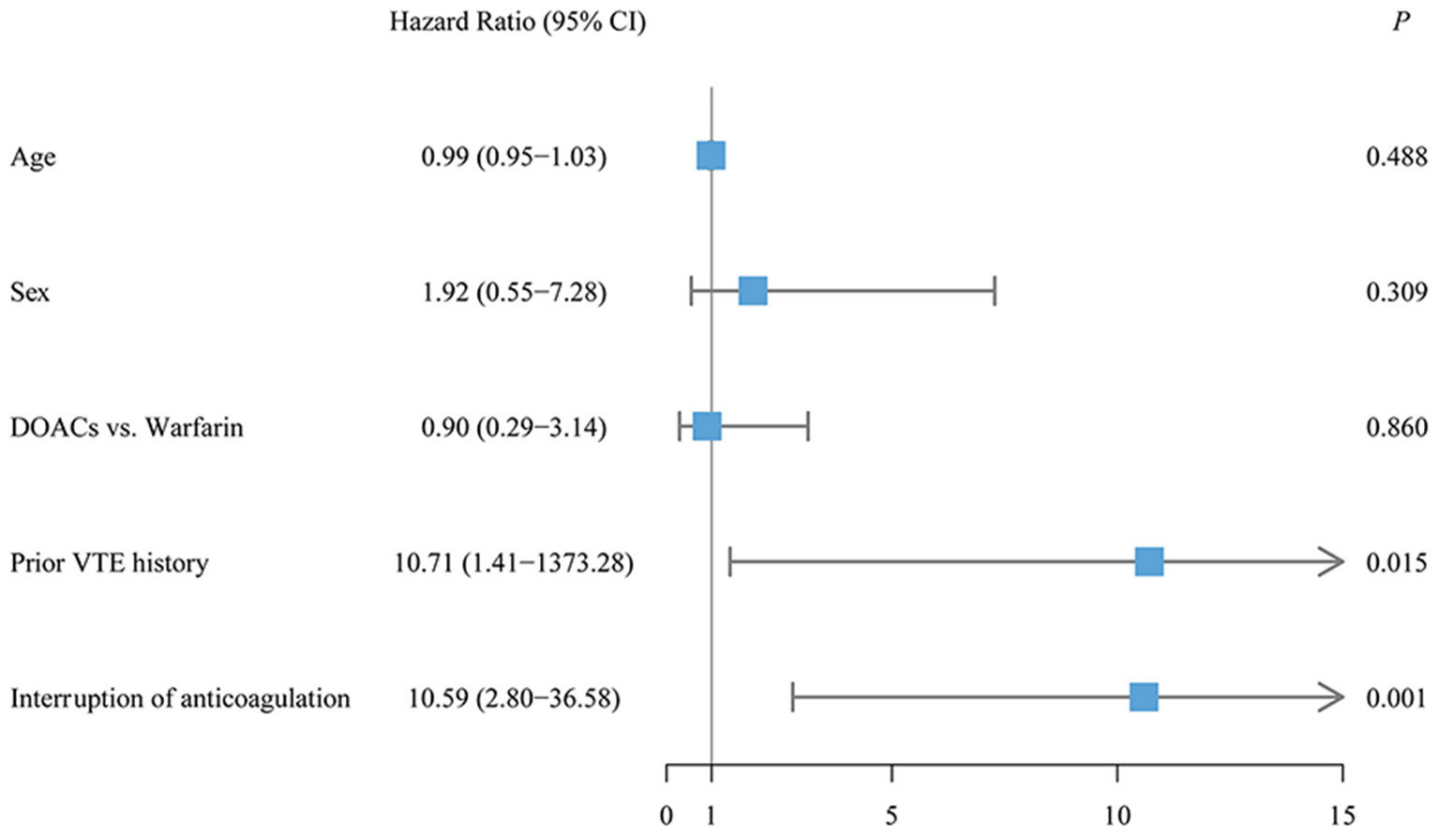
The multivariate Cox regression analysis for recurrent venous thromboembolism (VTE).

**Figure 4:**
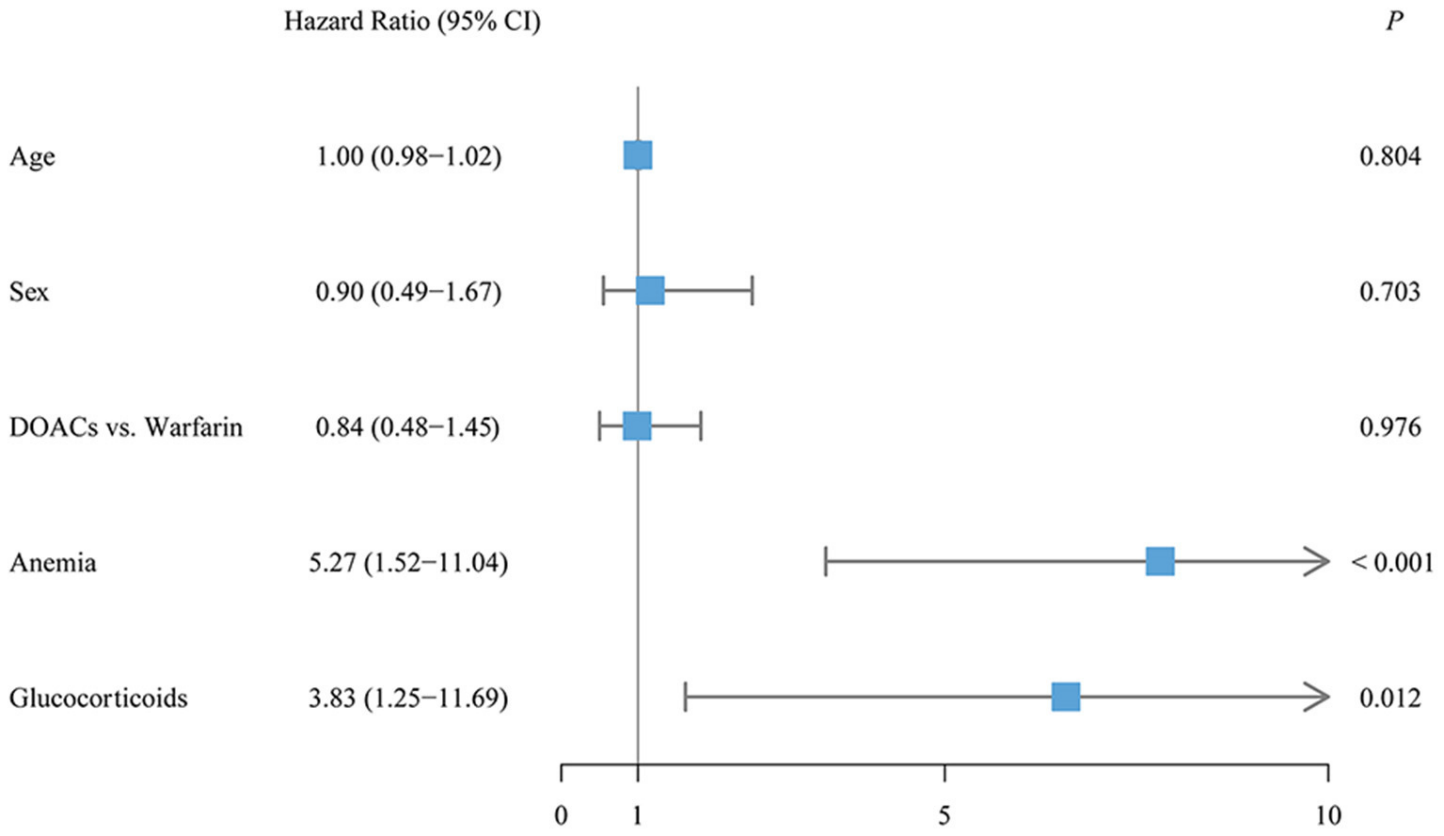
The multivariate Cox regression analysis for clinically relevant bleeding.

Risk factors associated with outcome events were assessed by the Cox proportional regression model. Prior VTE history (adjusted HR, 10.71; 95% CI, 1.41–1373.28; *P* = 0.015) and the interruption of anticoagulation treatment (adjusted HR, 10.59; 95% CI, 2.80–36.58; *P* = 0.001) were significantly associated with an increased risk of recurrent VTE in patients with CTEPH (Figure 3). Moreover, anemia (adjusted HR, 5.27; 95% CI, 1.52–11.04; *P* < 0.001) and glucocorticoid use (adjusted HR, 3.83; 95% CI, 1.25–11.69; *P* = 0.012) were significantly associated with a higher risk of clinically relevant bleeding (Figure 4). Sensitivity analyses did not materially change our findings after excluding the patients treated with apixaban (Supplementary Table 4).

## Discussion

Among CTEPH patients undergoing long-term anticoagulation treatment, we present the first clinical evidence of a lower recurrent VTE risk but a similar clinically relevant bleeding risk in patients without prior VTE history compared with those with prior VTE history. We further identified a significant association between a prior VTE history and interruption of anticoagulation treatment with an increased risk of recurrent VTE, as well as a significant association between anemia and glucocorticoid use with higher clinically relevant bleeding risk in CTEPH patients.

Recurrent VTE in CTEPH patients is one of the most important concerns in anticoagulation management. Only two studies, however, have reported the incidence of recurrent VTE during anticoagulation treatment in CTEPH patients so far [1.2/100 person-year in a Japanese cohort (6) and 0.76/100 person-year in a British post-PEA cohort (8)]. The incidence of recurrent VTE in our cohort was 1.69/100 person-year, similar to previous studies. All those data suggested that the incidence of recurrent VTE in CTEPH patients seemed to be slightly lower than that in patients with VTE undergoing extended anticoagulation treatment (ranging from 2.75 to 3.10/100 person-year) (13, 14). The finding could be partially explained by the low incidence of recurrent VTE in CTEPH patients without prior VTE history. Additionally, the median timespan of 19.3 months before the occurrence of the first recurrent VTE in our cohort is much longer than that reported in the British post-PEA cohort (5.8 months). The reasons for earlier recurrent VTE in the post-PEA cohort are still unknown and might be attributed to more frequent in-clinic visits after PEA surgery with near one-third of recurrent VTE being incidental findings on routine post-operative imaging in asymptomatic individuals (8).

Bleeding is another important concern during life-long anticoagulation in CTEPH patients. Previous studies reported major bleeding incidences during anticoagulation treatment in CTPEH patients varying from 0.67/100 person-year to 5.0/100 person-year (6–8). In this study, the incidence of major bleeding seems to be similar to those in previous reports on patients with VTE (13, 14). Meanwhile, the sites and kinds of bleeding in our CTEPH patients are also similar to those in the VTE population, excluding hemoptysis. In our cohort and the Japanese cohort (6), hemoptysis was the most common bleeding event during anticoagulation treatment and accounted for 34% (16 of 47 events) and 29% (6 of 21 events) of the total bleeding, respectively. It needs to be emphasized that hemoptysis is more closely related to the severity of PH itself than to the anticoagulation treatment (5). Therefore, hemoptysis could usually be alleviated by effective treatment for PH.

The absence of VTE history in approximately one-fourth of CTEPH patients has been reported previously (1). Instead of simply unresolved PE, the underlying explanations in those patients could be that they had recurrent “silent PE” (15) or group 1 pulmonary arterial hypertension with a pronounced thrombotic phenotype (16). Additionally, the alteration of fibrinogen structure and dysregulation of vWF axis have been investigated in CTEPH patients (17, 18). These disputations emphasized the significance of a multidisciplinary team from an expert center to confirm the diagnosis of CTEPH. Different from the typically segmental lesions in CTEPH, pulmonary perfusion scans usually show non-segmental defects or are normal in primary PH (19). Some features observed on CT pulmonary angiography including mosaic attenuation in the lungs, peripheral parenchymal infarcts, and irregular vessel size might also favor the diagnosis of CTEPH (20).

To the best of our knowledge, the present study is the first to report that CTEPH patients with a prior history of VTE are more likely to have recurrent VTE than those without a prior history of VTE, which is consistent with previous studies showing that patients with a prior history of VTE had an increased risk of recurrent VTE following surgery than those without a history of VTE (21–23). This result suggests that in CTEPH patients with a prior history of VTE, more attention should be paid to reducing the risk of recurrent VTE. Anticoagulants may also play an important role in the occurrence of recurrent VTE. In the present study, the patients without prior VTE were more often treated with warfarin than those with a prior history of VTE, which may have resulted in a lower risk of recurrent VTE. However, the result of multivariate Cox models with adjustment for anticoagulation treatment did not change the conclusion.

Apart from a prior history of VTE, interruption of anticoagulation treatment was also associated with a higher risk of recurrent VTE, and the finding corroborated previous research in an atrial fibrillation population (24). This phenomenon might be explained by the following: First, the interruption or withdrawal of anticoagulants has been mainly in response to bleeding in clinical practice. The rate of bleeding-related interruption is 68.8% (11/16) in the current study (Supplementary Table 1). Second, an interesting fact is that bleeding and thrombosis events share many common risk factors, resulting in patients with a high risk of bleeding probably also have a high risk of thrombosis (25, 26).

We identified anemia and glucocorticoid use being associated with an increased risk of clinically relevant bleeding. Anemia is a powerful predictor of bleeding during anticoagulation treatment across varying thrombotic diseases, with a prevalence of 33% in VTE (27), 14% in atrial fibrillation (28), and 14.5% in acute coronary syndrome (29). In our cohort, 28 (8.4%) CTEPH patients were anemic, of which seven patients had prior major bleeding. We speculated that anemia may reflect a recent subclinical bleeding or a predisposition to bleeding, thus contributing to an increased risk of bleeding. Bleeding, especially gastrointestinal bleeding, is a well-recognized side effect of glucocorticoid treatment. In this study, one-third of glucocorticoid-related bleeding in CTEPH patients was gastrointestinal bleeding (three of nine). As in the VTE population (27, 30), concomitant glucocorticoid use is also an important risk factor for bleeding in CTEPH patients.

The current study has several limitations. First, the retrospective nature of this study may introduce recall and measurement biases. Particularly, it is difficult for us to determine whether the patients had a prior history of VTE. However, this would be less likely to bias our findings considering that the prevalence of a prior history of VTE in this study is comparable to that in previous publications (1, 31). Second, the sample size of patients without a prior history of VTE was relatively small. Therefore, the interpretations regarding the anticoagulation outcomes in CTEPH patients without a prior history of VTE should be generalized with caution. Further prospective studies with larger sample size are warranted to verify the current findings. Finally, as asymptomatic VTE is not rare in clinical practice, our study may underestimate the real recurrent rate of VTE events. Nevertheless, regular screening for asymptomatic VTE in all CTEPH patients seems to be unnecessary and impractical.

## Conclusion

The present study is the first to reveal that a prior history of VTE significantly increases the risk of recurrent VTE in CTEPH patients during anticoagulation treatment. This finding should be further evaluated in prospective studies.

## Data Availability Statement

The raw data supporting the conclusions of this article will be made available by the authors, without undue reservation.

## Ethics Statement

The studies involving human participants were reviewed and approved by The Institutional Ethics Review Board of FuWai Hospital. The patients/participants provided their written informed consent to participate in this study.

## Author Contributions

Z-CJ and XJ conceived and designed the study. Y-JZ, Y-PZ, Y-PW, X-JZ, and Z-YL collected the baseline data. X-QX, X-XY, CL, and LH conducted the follow-up of patients. Y-JZ and KS performed statistical analysis. Y-JZ and XJ drafted the manuscript. All authors revised the manuscript for important intellectual content and approved the final version.

## Conflict of Interest

The authors declare that the research was conducted in the absence of any commercial or financial relationships that could be construed as a potential conflict of interest.
