# Supplementary material for: Association Between Anticoagulation Outcomes and Venous Thromboembolism History in Chronic Thromboembolic Pulmonary Hypertension
Source: Front Cardiovasc Med. 2021 May 21;8:628284. doi: 10.3389/fcvm.2021.628284 (PMC8175786; doi:10.3389/fcvm.2021.628284)
Supplement: Supplementary file 1 [file Data_Sheet_1.docx]

**Supplementary Material**

**Association between Anticoagulation Outcomes and Venous Thromboembolism History in Chronic Thromboembolic Pulmonary Hypertension**

*Yong-Jian Zhu, Yu-Ping Zhou, Yun-Peng Wei, Xi-Qi Xu, Xin-Xin Yan, Chao Liu, Xi-Jie Zhu, Zi-Yi Liu, Kai Sun, Lu Hua, Xin Jiang, Zhi-Cheng Jing*

## Supplementary Figures


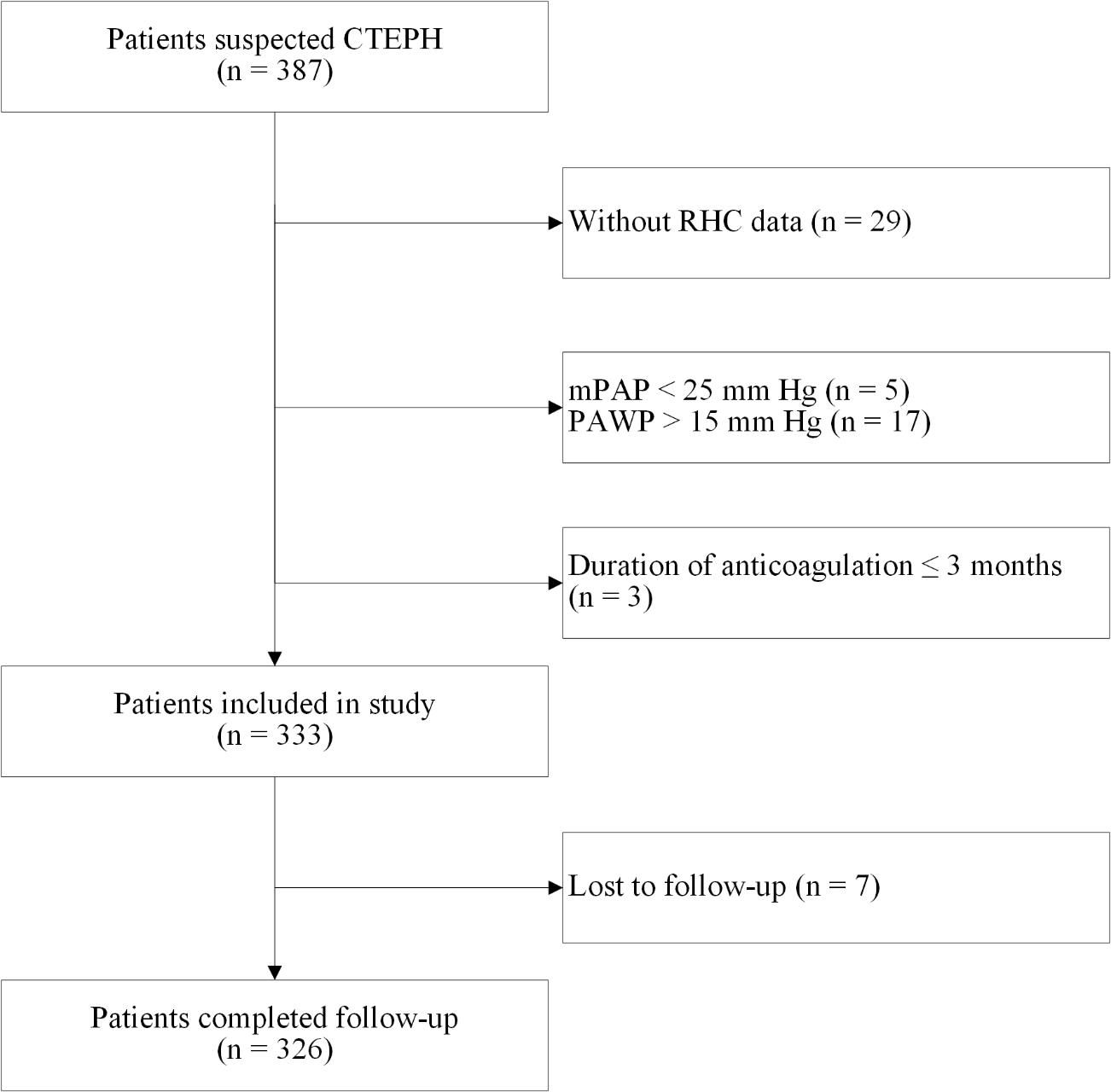


**Supplementary Figure 1.** Study flowchart**.**

CTEPH, chronic thromboembolic pulmonary hypertension; RHC, right heart catheterization; mPAP, mean pulmonary arterial pressure; PAWP, pulmonary arterial wedge pressure.


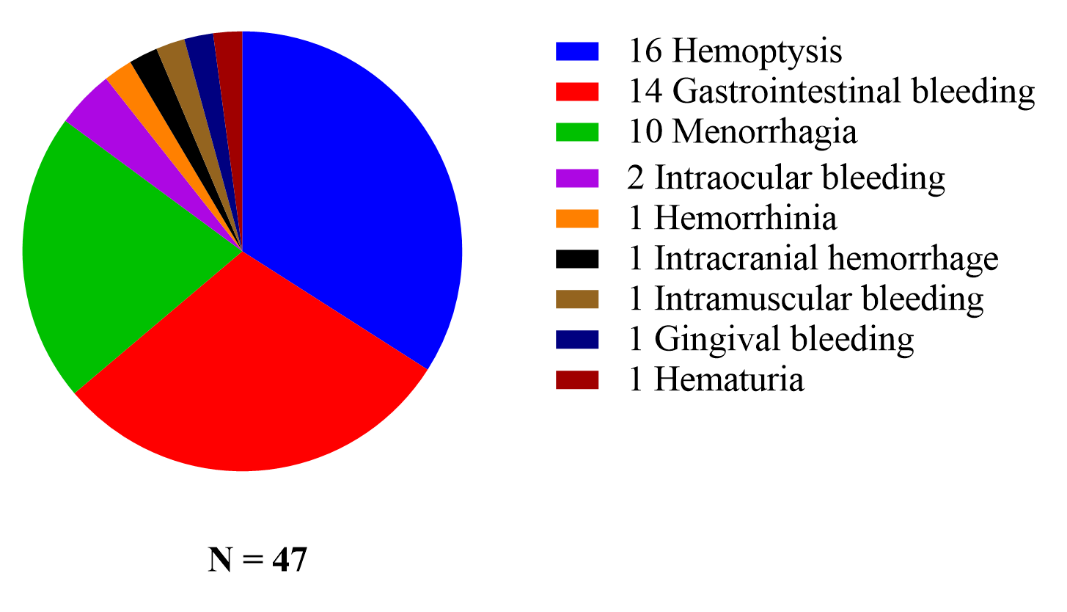


**Supplementary Figure 2.** The proportions of various hemorrhagic sites of clinically relevant bleeding.

## Supplementary Tables

**Supplementary Table 1.** The details of anticoagulants use.

|  | **n (%)** |
| --- | --- |
| Anticoagulants at diagnosis |  |
| Rivaroxaban, n (%) | 236 (70.9) |
| Dabigatran, n (%) | 9 (2.7) |
| Apixaban, n (%) | 2 (0.6) |
| Warfarin, n (%) | 86 (25.8) |
| Anticoagulants after discharge |  |
| Rivaroxaban, n (%) | 200 (60.1) |
| Dabigatran, n (%) | 3 (0.9) |
| Warfarin, n (%) | 89 (26.7) |
| Interruption of anticoagulants |  |
| Bleeding related, n (%) | 11 (3.3) |
| At discretion, n (%) | 5 (1.5) |
| Withdrawal of anticoagulants |  |
| Bleeding related, n (%) | 2 (0.6) |
| At discretion, n (%) | 9 (2.7) |

**Supplementary Table 2.** The univariate Cox regression analyses for recurrent venous thromboembolism.

|  | **HR (95% CI)** | ***P* Value** |
| --- | --- | --- |
| Age, years | 1.01 (0.97-1.05) | 0.715 |
| Female sex | 2.27 (0.70-7.38) | 0.173 |
| Blood group O | 2.98 (0.97-9.20) | 0.058 |
| Prior VTE history^*^ | 8.92 (1.18-1142.00) | 0.029 |
| APS | 1.26 (0.16-9.78) | 0.823 |
| Active cancer^*^ | 6.00 (0.05-46.10) | 0.332 |
| Renal insufficient^*^ | 1.46 (0.01-11.18) | 0.805 |
| Ischemic stroke | 0.77 (0.10-6.23) | 0.805 |
| Hypertension | 0.83 (0.23-3.08) | 0.782 |
| Diabetes mellitus | 2.25 (0.29-17.45) | 0.439 |
| D-dimer, ng/ml | 0.87 (0.58-1.31) | 0.511 |
| DOACs vs. Warfarin | 1.71 (0.47-6.29) | 0.420 |
| PH-targeted drugs^*^ | 1.87(0.25-239.65) | 0.632 |
| Interruption of anticoagulation | 12.70 (3.81-42.29) | < 0.001 |
| ^*^Penalized likelihood approach.  CTEPH, chronic thromboembolic pulmonary hypertension; VTE, venous thromboembolism; APS, antiphospholipid syndrome; DOACs, direct oral anticoagulants; PH, pulmonary hypertension. | | |

**Supplementary Table 3.** The univariate Cox regression analyses for clinically relevant bleeding.

|  | **HR (95% CI)** | ***P* Value** |
| --- | --- | --- |
| Age, years | 0.99 (0.97-1.01) | 0.445 |
| Female sex | 1.53 (0.76-3.08) | 0.239 |
| Blood group O | 1.49 (0.67-3.30) | 0.329 |
| Prior VTE history | 0.83 (0.38-1.78) | 0.623 |
| APS | 2.25 (0.78-6.46) | 0.132 |
| Renal insufficient^*^ | 1.87 (0.25-239.65) | 0.632 |
| Ischemic stroke | 1.00 (0.30-3.33) | 0.998 |
| Hypertension | 1.05 (0.49-2.26) | 0.909 |
| Diabetes mellitus | 0.77 (0.11-5.65) | 0.797 |
| Anemia | 5.25 (2.48-11.10) | < 0.001 |
| Log_NT-proBNP_ | 1.16 (0.66-2.04) | 0.605 |
| mPAP, mm Hg | 1.00 (0.97-1.02) | 0.713 |
| PVR, Wood units | 0.99 (0.92-1.07) | 0.855 |
| DOACs vs. Warfarin | 0.72 (0.35-1.47) | 0.367 |
| PH-targeted drugs | 0.99 (0.24-4.14) | 0.986 |
| Glucocorticoid use | 3.71 (1.29-10.65) | 0.015 |
| ^*^Penalized likelihood approach.  CTEPH, chronic thromboembolic pulmonary hypertension; VTE, venous thromboembolism; APS, antiphospholipid syndrome; NT-proBNP, N-terminal pro-B-type natriuretic peptide; mPAP, mean pulmonary artery pressure; PVR, pulmonary vascular resistance; DOACs, direct oral anticoagulants; PH, pulmonary hypertension. | | |

**Supplementary Table 4.** The multivariate Cox regression analyses after excluding patients treated with apixaban: sensitivity analyses.

|  | **HR (95% CI)** | ***P* Value** |
| --- | --- | --- |
| **Recent VTE^*^** |  |  |
| Age, years | 0.99 (0.96-1.03) | 0.640 |
| Female sex | 1.79 (0.53-6.66) | 0.348 |
| DOACs vs. Warfarin | 1.13 (0.35-4.60) | 0.843 |
| Prior VTE history | 10.44 (1.36-1341.34) | 0.018 |
| Interruption of anticoagulation | 12.63 (3.28-44.37) | < 0.001 |
| **Clinically relevant bleeding** |  |  |
| Age, years | 1.00 (0.98-1.02) | 0.948 |
| Female sex | 1.48 (0.72-3.06) | 0.290 |
| DOACs vs. Warfarin | 0.66 (0.32-1.39) | 0.273 |
| Anemia | 5.97 (2.74-13.04) | < 0.001 |
| Glucocorticoid use | 5.24 (1.67-16.47) | 0.005 |
| ^*^Penalized likelihood approach.  VTE, venous thromboembolism; DOACs, direct oral anticoagulants. | | |
